# Supplementary material for: Natural genetic variation in GLK1-mediated photosynthetic acclimation in response to light
Source: BMC Plant Biol. 2024 Feb 5;24:87. doi: 10.1186/s12870-024-04741-1 (PMC10840168; doi:10.1186/s12870-024-04741-1)
Supplement: Supplementary file 2 — Additional file 2: Suppl. Fig 1. Spatial distribution of the studied Arabidopsis accessions. A) World map plotting the location from where accessions were collected. A1 accessions are plotted in blue, A2 in red, and A3 in green. B) Proportion of country of origin is plotted for accessions belonging to clusters A1 and A2. C) Proportion of admixture groups as identified by the 1001 Genomes project [30]. Suppl. Fig 2. GO analysis among genes classified in the G1 and G2 groups. GO analysis was performed with ShinyGO v0.77. The GO (Biological processes) enrichment for G1 genes is shown in A, and for G2 genes is shown in B. ShinyGO can also test the enrichment among the Plant Gene Set Annotation database [51] and other databases. The enrichment using all databases in ShinyGO v 0.77 for G1 genes is shown in C and for G2 genes in D. Suppl. Figure 3. Expression of known GLK1 regulators in different Arabidopsis accession groups. The expression distribution of GLK2 (A), HY5 (B), PIF4 (C) and GUN1 (D) is shown for each group of accessions defined in Fig. 1a. Suppl. Figure 4. A Distribution of -log 10 p-values obtained by PLINK when testing (Chi-square test) for association between the group of accessions A1 versus A2 for each chromosome. Typically, a fixed p-value threshold of 5*-8 is widely used to identify association between genetic variants and a trait of interest [52]. B Quantile-Quantile (Q-Q) plot of the observed versus expected p-values. Red dashed line indicates the diagonal. Suppl. Figure 5. Predicted gene regulatory network around GLK1 estimated for the A1 group of accessions as described for Fig. 4A. Suppl. Figure 6. A Relation of GLK1 and BIA2 expression, as well as impact of mutation causing stop codon in the BIA2 gene. B Expression signature of accessions with high BIA2 expression fits with the observed consequence of BIA2 overexpression in Col-0. [file 12870_2024_4741_MOESM2_ESM.docx]

**Supplementary Figures**


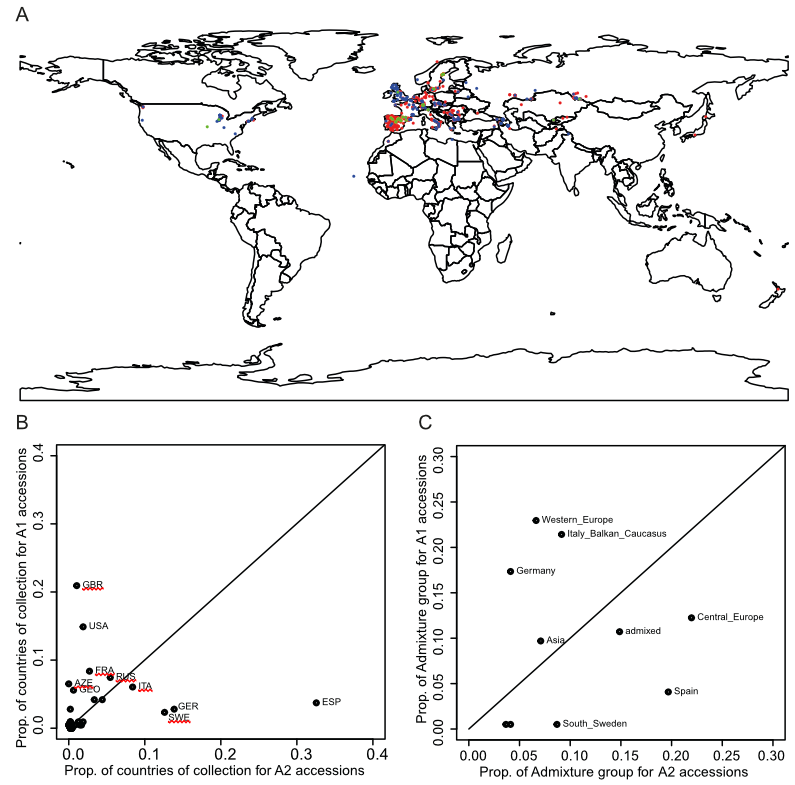


**Suppl. Fig 1. Spatial distribution of the studied *Arabidopsis* accessions.** **A)** World map plotting the location from where accessions were collected. A1 accessions are plotted in blue, A2 in red, and A3 in green. **B)** Proportion of country of origin is plotted for accessions belonging to clusters A1 and A2. **C)** Proportion of admixture groups as identified by the 1001 Genomes project [30].


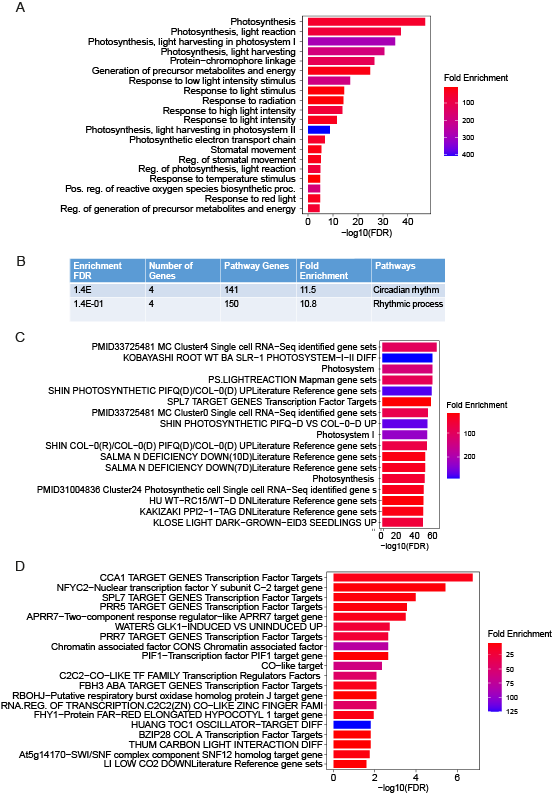


**Suppl. Fig 2. GO analysis among genes classified in the G1 and G2 groups.** GO analysis was performed with ShinyGO v0.77. The GO (Biological processes) enrichment for G1 genes is shown in **A**, and for G2 genes is shown in **B**. ShinyGO can also test the enrichment among the Plant Gene Set Annotation database [51] and other databases. The enrichment using all databases in ShinyGO v 0.77 for G1 genes is shown in **C** and for G2 genes in **D**.


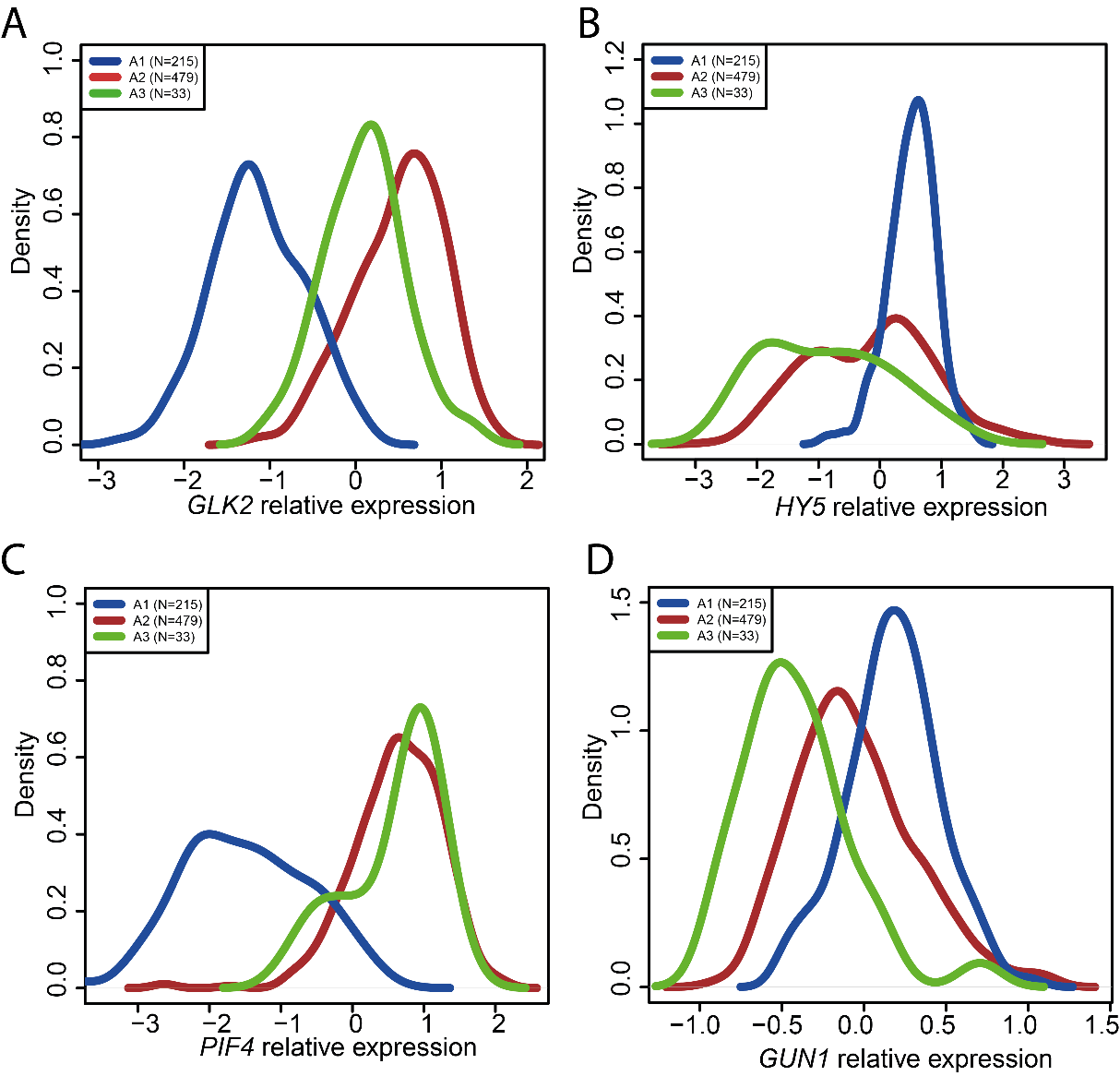


**Suppl. Figure 3. Expression of known *GLK1* regulators in different *Arabidopsis* accession groups.** The expression distribution of *GLK2* (A), *HY5* (B), *PIF4* (C) and *GUN1* (D) is shown for each group of accessions defined in Fig. 1a.


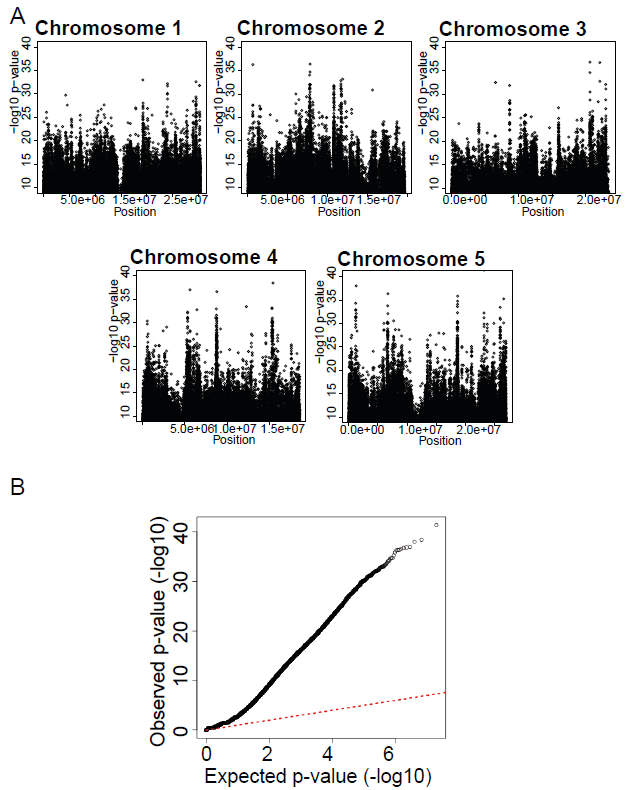


**Suppl. Figure 4.** **A** Distribution of -log 10 p-values obtained by PLINK when testing (Chi-square test) for association between the group of accessions A1 versus A2 for each chromosome. Typically, a fixed p-value threshold of 5*-8 is widely used to identify association between genetic variants and a trait of interest [52]. **B** Quantile-Quantile (Q-Q) plot of the observed versus expected p-values. Red dashed line indicates the diagonal.


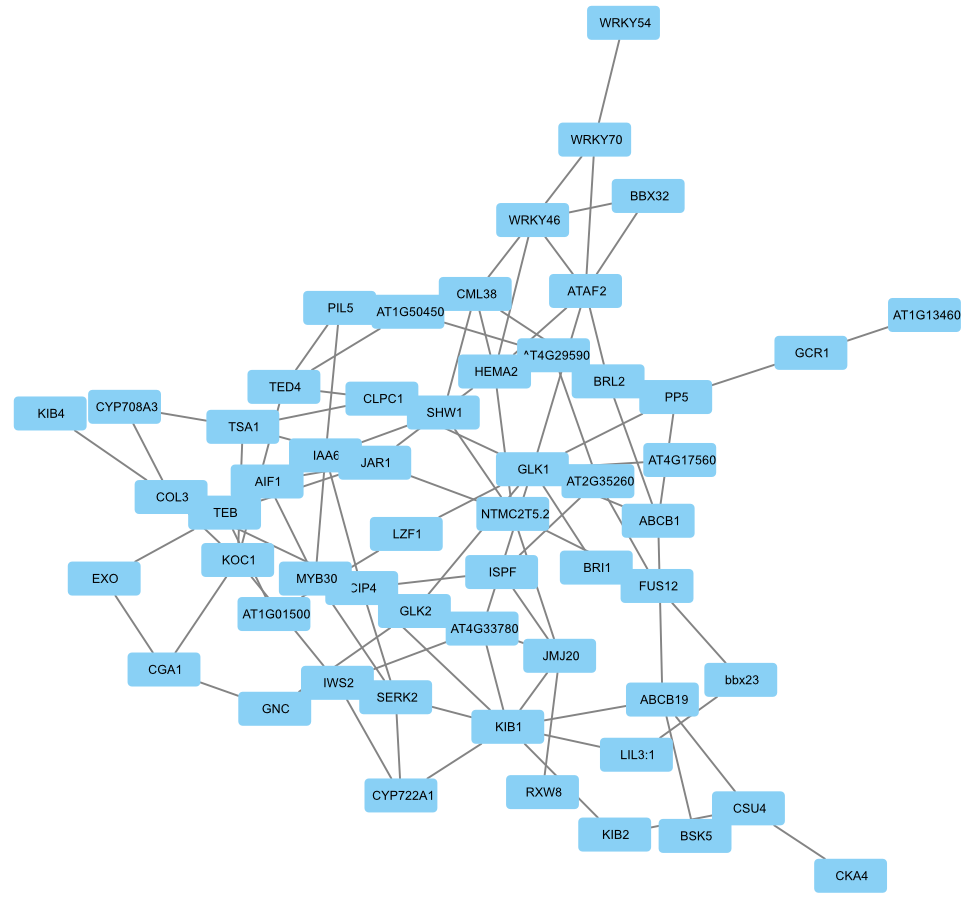


**Suppl. Figure 5.** Predicted gene regulatory network around GLK1 estimated for the A1 group of accessions as described for Fig. 4A.


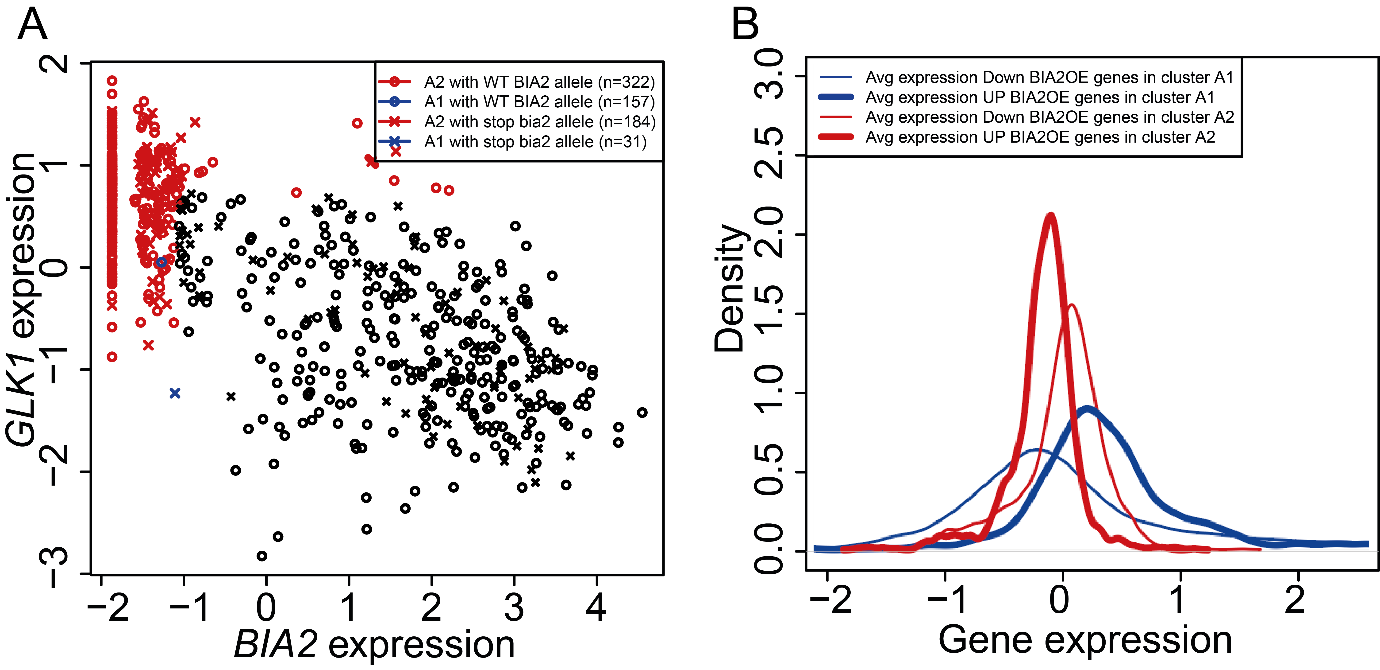


**Suppl. Figure 6. A** Relation of *GLK1* and *BIA2* expression, as well as impact of mutation causing stop codon in the *BIA2* gene. **B** Expression signature of accessions with high *BIA2* expression fits with the observed consequence of *BIA2* overexpression in *Col*-0.

**References (specific to Supplement)**

51. Ma X, Yan H, Yang J, Liu Y, Li Z, Sheng M, et al. PlantGSAD: a comprehensive gene set annotation database for plant species. Nucleic Acids Res. 2022;50:D1456–67.

52. Chen Z, Boehnke M, Wen X, Mukherjee B. Revisiting the genome-wide significance threshold for common variant GWAS. G3 Genes, Genomes, Genet. 2021;11.

**Supplementary Tables**

**Table S1:** List of gene TAIR IDs in clusters G1, G2 and G3.

**Table S2:** List of Arabidopsis natural accessions for clusters A1, A2 and A3.

**Table S3:** Mean difference (t-test) between the environmental variables (WorldClim 2.0) of the place of collection of the accession cluster A1 versus A2

**Table S4:** Different association (Chi-square) of genetic variants between cluster A1 versus A2

**Table S5:** Gene regulatory network estimated by Genenet for accession cluster A1.

**Table S6:** Gene regulatory network estimated by Genenet for accession cluster A2.
